# Supplementary material for: 16S rRNA gene sequencing of rectal swab in patients affected by COVID-19
Source: PLoS One. 2021 Feb 17;16(2):e0247041. doi: 10.1371/journal.pone.0247041 (PMC7888592; doi:10.1371/journal.pone.0247041)
Supplement: Attachment — Submitted filename: Response to Reviewers.docx [file pone.0247041.s014.docx]

Review Comments to the Author

**Reviewer #1:** The authors have performed a pilot study examining the gut microbiomes of patients admitted to the ICU or infectious disease ward at their institution with or without COVID19-associated pneumonia. The data support a loss of bacterial diversity in COVID-positive patients and gain of potentially pathological species.

Revisions I think should be made before publication include:

1) The methods are too vague.

a. For example, was the PCOA performed in GAIA? What about the Venn Diagrams, etc.? It's not stated, nor for most of the other figures.

RE: We thank the reviewer for this comment and we apologize for the missing information. We have now provided more details about the methods in the respective paragraph.

b. How were the different bacterial profiles generated by the different hypervariable regions combined? How did they differ?

RE: We apologize for the lack of clarity. The sequencing run has generated in total 16x10^^6^ reads with the 77% of high quality reads (21% low quality, 2% test fragments). Finally we obtained 5.5x10^^5^ reads per sample (reads length were 244 bp mean, 260 bp median and 289 bp mode) and all analyzed specimens showed a suitable library’s profile. The analysis was performed by 16S Metagenomics GAIA 2.0 software and DESeq2 package software. Sequence data generated as FASTQ files, were analysed using the 16S Metagenomics GAIA 2.0 software which performs the quality control of the reads/pairs (i.e., trimming, clipping and adapter removal steps) through FastQC and BBDuk. The reads/pairs are mapped with BWA-MEM against the 16S databases (based on NCBI). Differential expression analysis using DESeq2 package to test for differential analysis by use of negative binomial generalized linear models was used. Only changes with FDR below 0.05 were considered significant.

c. How many reads were generated per patient? Was this sufficient to capture the long tail of the gut microbiomes?

RE: We thank the reviewer for this comment. We have obtained an average of 5.5 x 10^^5^ reads each sample. Reads were sufficient to evaluate optimal rarefaction curves as shown in the following graphics. Graphics were plotted based on Chao index, observed species, shannon and simpson index respectively. The rarefaction curves suggest that the samples have plateaued; this means that a good representation of the microbial community was obtained for the samples analysed in this study.

d. What was the similarity level used to bin reads and identify OTUs? If this was all done using default settings in GAIA, it should stated.

RE: All analyses are executed with default settings described in https://www.biorxiv.org/content/10.1101/804690v1. The analysis is amplicon-seq, so the identity threshold for species is 97%, for genus is 93%.

e. One hypervariable region is listed as V-2-4-8 which does not make sense, I think they should be listed as V2, V4, V8 to differentiate these single hypervariable region amplicons from the V3-6 amplicons which actually span hypervariable regions V3 through V6.

RE: We agree with the reviewer. We have now added these changes within the text.

f. which NCBI database was used?

RE: RE: 16S sequences available from GenBank with meaningful taxonomic information in at least the genus level (those classified as uncultured, unidentified, etc. were excluded). In addition, 16S sequences were rescued from entire genomes that also available from GenBank using the corresponding annotation.

2) The results lack quantitation. For example in lines 158-176 no fold changes in relative abundance are reported for the organisms listed. Nor is there discussion of the prevalence of these bacteria that may constitute potential biomarkers of COVID infection. For example, In supplemental table 1, Spirochaetes and Fusobacteria were found to be significantly down in ward patients relative to control, but neither Phylum is prevalent enough to even be visible in Figure S1. Are these results just noise from very rare organisms that may or may not be found at the sampling depth of the study? While the statistics are this is not the case, and the authors have adjusted for multiple testing (FDR p-values) some discussion of the low prevalence of the significantly altered organisms would make the findings more convincing.

RE: We thank the reviewer for this comment. Since numerous changes were observed, we preferred to report p values, FDR score and fold changes in the supplementary tables instead of reporting these values within the manuscript which would have been difficult to read with all the number inserted. We have now added more sentences in order to better describe the changes observed tanking in consideration also the comment below.

Another way to look at my concern here is to consider why it is the ward and ICU patients have differences in their affected bacterial families in lines 158-168 (while also having similarities) when both cohorts have COVID infection, If the families listed were important to COVID infection then they should be found in both ward and ICU patients. Either there is an affect of ward versus ICU which should be discussed or these families are noise in the data despite passing statistical muster.

RE: We thank the reviewer for this insightful comment. As stated in our manuscript, this is a pilot study that represent preliminary data and we are aware that the patient’s number is limited. We cannot exclude that increasing the patients number to be enrolled it is possible to discover more families having similarities in both groups. Nevertheless, several families in common were linked to the virus presence independently of the ward in which the patients are hospitalized. Furthermore, the severity disease and the intestinal inflammation state were different so the variability expressed at the family level is higher in ICU patients in comparison to those observed in patients hospitalized in the ward as also reported by Ravi et al (PMID: 31526447). Our intent was to describe the common and the exclusive microorganisms belonging to the different groups. We agree that families listed due to only COVID infection (either ward or ICU) are worth of note and for this reason we reported that when considering the i-COVID19 as compared to CTRL, in addition to some bacteria in common with w-COVID19 patients (i.e. *Staphylococcaceae, Aerococcaceae, Dermabacteraceae, Actinobacteria* and so on Fig.4, and Fig. 6A and Supporting material S5 table) *Erysipelotrichaceae, Microbacteriaceae, Mycobacteriaceae, Pseudonocardiaceae, Brevibacteriaceae*, and others reported in Supporting material S5 table were significantly increased while *Carnobacteriaceae, Coriobacteriaceae* and *Mycoplasmataceae* were significantly reduced.

But we also underlined the exclusive microorganisms belonging to the two distinct groups: *Staphylococcaceae, Microbacteriaceae, Micrococcaceae, Pseudonocardiaceae, Erysipelotrichales* and others reported in Supporting material S6 table were significantly higher in i-COVID-19 as compared to w-COVID19. *Carnobacteriaceae, Pectobacteriaceae, Moritellaceae, Selenomonadaceae, Micromonosporaceae, Coriobacteriaceae* and few others were significantly decreased in i-COVID19 as compared to w-COVID19.

We then discussed all these changes considering the already known microorganisms reported within the literature.

3) There appears to be significant variation between patients within the three main categories (ward, ICU, control). For example, in supplementary figure 1 there is one control and one ward patient with surprisingly high Proteobacteria and low Firmicutes/Bacteriodetes. In addition, one ward patient apparently has no Bacteriodetes at all - a shocking result given this is typically the major Phylum on gut microbiomes. There should be some discussion of the variation and it's potential to affect the development of potential biomarkers.

RE: We thank the reviewer for this comment. We noticed also these changes and we discussed what we thought were the most important. However, in agreement with the reviewer comment we have now added some discussion on the above mentioned changes.

4) Some discussion of why there was a significant difference in Ferritin in the ICU patients would be helpful for a non-clinical audience.

RE: We thank the reviewer for this comment. We better clarified the concept with the following sentences reported in the manuscript: ‘Moreover, ANOVA and Kruskal-Wallis analysis reveal that the variables between our patient cohorts was not noteworthy except for ferritin level that is significantly lower in ICU patients.

Ferritin is a marker of inflammation and the high levels of ferritin detected in i-COVID19 patients in comparison to w-COVID19, may be associate with a greater severity of the disease and adverse outcomes. Normally, ferritin is able to activate macrophages that when stimulated begin to secrete cytokines that at low concentrations, help to protect the body from viruses and bacteria. On the other hand, high levels of ferritin activate more macrophages that produce the so-called "cytokine storm" which can be lethal for the body’ (PMID: 32268212).

Minor changes:

1) Figure S1 the type is difficult to read because of low resolution, try to use vector type rather than rasterized type.

RE: We regret that the figures are not visible, we have carefully checked all of the figures and the resolution is 300 DPI or more, as requested, we hope to the reviewer’s satisfaction. We trust that we have now resolved the issues pertaining to poor resolution, which can be also resolved by accessing the access/download high-resolution link for each image provided within the text

2) Figure S2 the legend is cut of on the right side making it impossible, for example, to identify the Fusobacteria. The type is also nearly unreadable due to rasterization.

RE: We trust that we have now resolved the issues

3) Figure 5 bacterial names are unreadable due to low resolution.

RE: We regret that the figures are not visible, we have carefully checked all of the figures and the resolution is 300 DPI or more, as requested, we hope to the reviewer’s satisfaction. We trust that we have now resolved the issues pertaining to poor resolution which can be also resolved by accessing the access/download high-resolution link for each image provided within the text

4) line 36 replace remarkably with remarkable; 5) line 46 replace different with several; 6) line 67 replace examined with performed; 7) line 138 replace resulted with was; 8) line 150, 155 insert the between At and Phylum; 9) line 150,153 replace resulted with were; 10) line 158 insert the between At and Family; 11) line 218, 225 replace decreased with decrease;12) line 229 the phrase "it popped out" is awkward and not formal English for a publication; 13) line 232 the first sentence would read better if simply changed to "There are several limitations to this study."; 14) insert a between administered and few; 15) line 247 replace study with studies.

RE: We thank the reviewer for these suggestions. We have now edited the text according to the reviewer’ comments.

**Reviewer #2:** PONE-D-20-29220 16S rRNA Gene Sequencing of Rectal Swab in Patients Affected by COVID-19

In the current study, the Researchers investigated whether COVID infection promoted alterations in the gut microbiome diversity and profiles, as well as whether these shifts conferred severity of the disease state. While the study is limited in subject enrollment (which can be overlooked since it is a pilot study), there are some potentially novel outcomes of the data sets provided. However, due to the vast limitations of the study, further details on methodologies and additional analyses are required. Furthermore, the authors should temper their conclusions drawn from the current data set, as the study design does not allow for determination that COVID infection mediated or promoted the observed outcomes.

RE: We thank the reviewer for the overall positive comment. We agree with the reviewer that since it is a pilot study conclusion should be tempered and for this reason, we have integrated a paragraph in the discussion section stating the limitations of our study.

Major concerns/comments:

1. The major concern of this Reviewer is that there is no "pre-COVID" data on the gut microbiome profiles of these patients, so it is impossible to definitively conclude that these microbiota profiles are due to the COVID infection. These profiles could be due to variation in diets, environment, medications, and/or existing comorbidities. At the very least this limitation needs to be addressed and the conclusions that these data provide evidence for COVID mediating these outcomes needs to be tempered.

RE: We agree with the reviewer that conclusion should be tempered and for this reason, we have integrated a paragraph in the discussion section stating the limitations of our study (line 236-257). We have only described a picture of what we observed in our patients without stating that the changes were correlated to any particular conditions since the study design did not allow us to do it.

2. The individual microbiota graphs need to be provided in the main text vs. as supplementary files. Furthermore, it appears that for most statistical endpoints reported in the group averages, only 1 or 2 individuals in each group drove those changes represented in the averages. For example Proteobacteria at the Phylum level are so varied, even amongst the controls, that it appears that the averages presented in Figures 3 and 4 are not necessarily representative of the whole group.

RE: We thank the reviewer for this insightful comment. We provide individual microbiota profile as supplementary figure 1 and 2 (at phylum and family levels respectively) and PCoA based on Bray-Curtis distance in order to be more exhaustive about the differences between the groups. We wish to underline that our aim was to display the unsupervised hierarchical analysis at the phylum and family level in order to reveal a characteristic “CTRL” segregated from those of COVID-19 positive patients (figure 5). Strikingly, a distinct profile can be distinguished between i-COVID19 and w-COVID19 with the latter being closer to CTRL as can be seen by the arrangements of the 3 groups. Definitely data interpretation should have considered true healthy controls for comparison, however we have added the figure 2 depicting the Principal Component analysis (PCoA) that displayed almost distinct clustering patterns among the three groups CTRL (red) w-COVID19 (green) and i-COVID19 (blue). Although we agree with the reviewer that for some Phyla and Families the levels are varied among the controls this can be due to the intrinsic inter-individual variability depending on several factors such as diet, genetic background, method of delivery at birth (https://www.ncbi.nlm.nih.gov/pmc/articles/PMC5483960/), infant feeding, physical activity, stress, medications and so on. Moreover, although it can be guessed that our control group could not being ideal, (3 recovered in ICU and 6 in ward), it allowed us to eliminate the variables within the microbiota profile of the hospitalized controls negative for COVID19.

3. In addition, information for each of the subjects should be correlated to their individual microbiome - this can be provided in a table in the supplemental files (e.g. Subject Control 1, age, sex, antibiotics, comorbidities). This is important, because it may allow for further distinction of effects of antibiotics and co-morbidities on the highly varied individual microbiome reads.

RE: We thank the reviewer for this comment. However, since it is a pilot study due to the small sample size we prefer to avoid the correlation with the different microbiota profiles in order to avoid misleading conclusions.

4. Further clarification on the analysis of the 16S sequencing needs to be provided. What were total reads, and were they comparable across all samples? Was the data rarefied for alpha and beta diversity analysis?

RE: We thank the reviewer for this comment. We have obtained an average of 5.5 x 10^^5^ each sample. Reads were sufficient to evaluate optimal rarefaction curves for alpha and beta diversity (16S Metagenomics GAIA 2.0 software) as shown in the following graphics. Graphics were plotted on the basis of Chao index, observed species, shannon and simpson index respectively. The rarefaction curves suggest that the samples have plateaued; this means that a good representation of the microbial community was obtained for the samples analysed in this study.

5. A more in-depth description of how samples were collected needs to be provided. One of the main concerns with microbiome analysis is minimizing contamination from other sources in your samples, which in this case would have been difficult. As such, can the authors rule out that the microbiota analyses do not include those microbiota on the surface of the skin vs. the actual GI tract? This is one of the main downfalls of using a rectal swab vs. fecal samples.

RE: We thank the reviewer for this comment. To collect the ideal sample is very difficult to obtain. Furthermore, as reported by Biehl et al., (PMID: 30986251) is not quite difficult to rule out if characteristic taxa (as *Corynebacterium, Staphylococcus, Streptococcus*) are representative of skin microbiota or if the high antibiotic burden and ongoing chemotherapy led to a shift towards these taxa in the distal intestinal microbiome. In any case, to minimize the 'external' contamination of our samples, we have used several prevention measures: 1) rectal swabs were performed one or two days after the hospitalization; 2) swabs surface were disinfected before to perform the analysis; 3) samples that were not analyzed immediately were stored at -80°C in a dedicate box; 4) swabs were collected using standardized and validated protocols for all patients; 5) a DNA automatic extractor were used to reduce the environmental contamination.

6. Were the patients that tested negative for COVID-19 (the controls) only tested 1 time?

RE: We thank the reviewer for this comment. We utilized different molecular methods and platform for SARS-CoV2 identification to be sure of the correct result. All patients included in our study (confirmed cases and negative controls) were tested for SARS-CoV2 several times. We analyzed multiple consecutive naso-pharyngeal swabs during the hospitalization.

7. Is the PCoA graph presented in Fig 2 weighted or unweighted?

RE: PCoA was not performed by UniFrac weighted or unweighted. Bray-Curtis dissimilarities were used. Gaia cannot calculate UniFrac distances but can perform PCoA based on Bray-Curtis dissimilarities. We have now better reported this information.

8. Analysis of Molecular Variance (AMOVA) and Analysis of similarities (ANOSIM) to assess the variations and similarities among different groups would be beneficial, especially considering the PCoA graph does not show distinct clustering for most groups.

RE: We agree with the reviewer that PCoA graph does not show very distinct clustering for most groups. This is because the high heterogeneity of the microbiota in every patients and the low number of cases considered as it is a pilot study. We performed a DeSeq analysis. The DESeq2 package provides methods to test for differential analysis by use of negative binomial generalized linear models. It is one of the leading methods of analysis due to its high sensitivity and low false discovery rate.

9. Additional alpha diversity analyses should be completed since only one of the chosen tests revealed statistical significance. The authors may want to consider abundance coverage-based estimator (ACE) richness and evenness analyses.

RE: We thank the reviewer for this comment. Unfortunately, this index is not calculated in IonReporter.

We reported many indices of alpha-diversity, among them the Shannon index, which considers both richness and evenness. Unfortunately, our software cannot calculate ACE.
