# Supplementary material for: 16S rRNA gene sequencing of rectal swab in patients affected by COVID-19
Source: PLoS One. 2021 Feb 17;16(2):e0247041. doi: 10.1371/journal.pone.0247041 (PMC7888592; doi:10.1371/journal.pone.0247041)
Supplement: Attachment — Submitted filename: Response to Reviewers.docx [file pone.0247041.s015.docx]

**Reviewer #1:** The authors have thoughtfully addressed most of the concerns of both reviewers, however, they have done so in the response to reviewers, and not in the manuscript. For example, both reviewers noted that the number of reads per sample was not reported which goes a long way to assuaging a knowledgeable reader's concerns about sequencing depth, especially when paired with the alpha diversity analysis also shown only in response to reviewers. The authors responded with the details that should be in the methods, but did not add them to the manuscript. Specifically to the point above, something like the following text:

"The sequencing run has generated in total 16x10^6 reads with the 77% of high quality reads (21% low quality, 2% test fragments). Finally we obtained 5.5x10^5 reads per sample (reads length were 244 bp mean, 260 bp median and 289 bp mode) and all analyzed specimens showed a suitable library’s profile. The analysis was performed by 16S Metagenomics GAIA 2.0 software and DESeq2 package software. Sequence data generated as FASTQ files, were analysed using the 16S Metagenomics GAIA 2.0 software which performs the quality control of the reads/pairs (i.e., trimming, clipping and adapter removal steps) through FastQC and BBDuk. The reads/pairs are mapped with BWA-MEM against the 16S databases (based on NCBI). Differential expression analysis using DESeq2 package to test for differential analysis by use of negative binomial generalized linear models was used. Only changes with FDR below 0.05 were considered significant" should be added to the methods, not just in the response to reviewers. Think of the reviewers as interested readers, if we both wanted this information, so will the readers. Similarly, the percent similarity used to determine species and genus calls, details on the reference sequence database used, etc. should be in the methods. In addition, it would be an improvement to include the CHAO1 alpha diversity plot in the response to reviewers in the supplemental figures. There is no word limit or figure limit in PLOS ONE, so there is no reason to leave out these details which provide the reader with key information to understand and trust the results.

Re: We apologize for missing to add this information within the text. We have now reported all the information contained in our previous response within the manuscript. The percent similarity used to determine species and genus calls was 93% at genus, 97% at species as reported in the new version of the text. Chao1 index is reported in figure 1.

In addition, it is still unclear how the analysis was performed on the reads, while the methods paragraph quoted above would suffice, later in the response to reviewers (reviewer 2, point 9) Ion Reporter is mentioned and strongly implies the reads were processed in some way on that platform. However, no mention of Ion Reporter is made in the manuscript. In addition, use of Ion Reporter would explain how the identity calls made from the multiple hypervariable regions were combined, but the authors still have not addressed this question in the response to reviewers or the manuscript (reviewer 1, point 1b).

RE: We apologize for the lack of clarity. Ion Reporter is one of the software that could be used for 16S analysis. However, Ion Reporter software was not mentioned in the manuscript because it is an analysis that has not been done for our sequences. For our whole study, we utilized the 16S Metagenomics GAIA 2.0 software and DESeq2 package software. Specifically, sequence data generated as FASTQ files, were analyzed using the 16S Metagenomics GAIA 2.0 software which performs the quality control of the reads/pairs (i.e., trimming, clipping and adapter removal steps) through FastQC and BBDuk. The reads/pairs are mapped with BWA-MEM against the 16S databases (based on NCBI). Differential expression analysis using DESeq2 package to test for differential analysis by use of negative binomial generalized linear models was used.

Ion Reporter was only mentioned in response to Reviewer 2 who asked us if it was possible to calculate the abundance coverage-based estimator (ACE) richness and evenness analyses.

Lastly, In response to reviewer 1, point 3 the authors state that discussion of the strikingly absence of Bacteriodetes in one patient has been added to the text, however, this reviewer cannot find it in the revised manuscript and a search of the revised manuscript for the word "Bacteriodetes" returned no results. If the text has indeed been added, the authors should refer to specific line numbers.

RE: We thank the reviewer for this comment. We already reported (line 231-235) that growing evidence has shown that perturbation of the gut microbial community may fuel blooms of otherwise low abundance and harmful bacteria which can further exacerbate the intestinal inflammation. Indeed, dysbiosis in the distal gut is often characterized by a decrease in the prevalence of strict anaerobes and an increased relative abundance of facultative anaerobic bacteria.

For these reasons now we add thisese sentences in the revised manuscript (line 228-231):’Notably, a clear case of profound dysbiosis (Fig. 5) was observed in one ward patient (90-year-old patient with diabetes, meningioma and osteoporosis in association with an increase of C-Reactive Protein (CRP) and lymphocytes), with a significant increase in Proteobacteria and a relevant reduction in Bacteriodetes, reflecting an important inflammatory state’.

**Reviewer #2:** While the authors have addressed many of the initial concerns in the revised manuscript, there are still some issues that need to be further addressed (some of which were in the initial major concerns/comments previously):

1. The Supplemental Figures S1 and S2 need to be moved to the main body of the manuscript, instead of being placed in the Supplemental files. These are extremely important figures for transparency of the major limitations of this study (small sample size, antibiotic use, no pre- vs. post data, etc.). The reader should not have to seek this information in the Supplemental Files, since they pertain to the main findings of the study, especially since individual variation seemed to drive the significant response in some of the reported outcomes within study groups.

RE: We thank the reviewer for this comment and we have now moved Figure S1 and S2 (now renamed Figure 5 and Figure 6) within the manuscript as suggested.

2. The authors should address in the Discussion (at a minimum) whether age, gender, co-morbidities, and/or antibiotic treatment (n=11 on study) altered individual 16S microbiota profiles reported.

RE: As suggested we have now added few sentences in the discussion section about the link between clinical data and the microbiota profiles. As for antibiotic treatment, we reported more details in lines 257-263. We recognize the potential effect of clinical/demographic data on the changes of mirobiota profile however we wish to underline that these data were not significantly different among the three groups.
